# Concentration variance decay during magma mixing: a volcanic chronometer

**DOI:** 10.1038/srep14225

**Published:** 2015-09-21

**Authors:** Diego Perugini, Cristina P. De Campos, Maurizio Petrelli, Donald B. Dingwell

**Affiliations:** 1 Department of Physics and Geology, University of Perugia, Piazza Università, 06100, Perugia, Italy; 2 Department of Earth and Environmental Sciences, Ludwig-Maximilians-Universität, München, 80333, Munich, Germany; 3 Department of Mineralogy and Geotectonics, University of São Paulo, Rua do Lago, USP, 05508-080, São Paulo, Brazil

## Abstract

The mixing of magmas is a common phenomenon in explosive eruptions. Concentration variance is a useful metric of this process and its decay (CVD) with time is an inevitable consequence during the progress of magma mixing. In order to calibrate this petrological/volcanological clock we have performed a time-series of high temperature experiments of magma mixing. The results of these experiments demonstrate that compositional variance decays exponentially with time. With this calibration the CVD rate (CVD-*R*) becomes a new geochronometer for the time lapse from initiation of mixing to eruption. The resultant novel technique is fully independent of the typically unknown advective history of mixing – a notorious uncertainty which plagues the application of many diffusional analyses of magmatic history. Using the calibrated CVD-*R* technique we have obtained mingling-to-eruption times for three explosive volcanic eruptions from Campi Flegrei (Italy) in the range of tens of minutes. These in turn imply ascent velocities of 5-8 meters per second. We anticipate the routine application of the CVD-*R* geochronometer to the eruptive products of active volcanoes in future in order to constrain typical “mixing to eruption” time lapses such that monitoring activities can be targeted at relevant timescales and signals during volcanic unrest.

The mobility of chemical elements in magmas plays a key role in the homogenization of the compositional gradients that attend the processes of magma differentiation. The action of chemical gradients and their homogenization in the melt phase influences, for example, the rates of crystallization, assimilation of country rocks, and magma mixing. The latter is a ubiquitous petrologic process involved in planetary evolution^1^,^2^,^3^,^4^,^5^,^6^. Magma mixing is also recognized to be a major process in generating extreme compositional variations in magmas^5^,^7^,^8^. It is also commonly associated with highly explosive volcanic eruptions^9^,^10^,^11^,^12^. Its detailed understanding is therefore of primary importance for petrology and volcanology, with direct implications for volcanic monitoring during volcano unrest, and as a consequence, for hazard mitigation planning and risk analysis.

In principle the rate of homogenization of compositional gradients in natural melts can be quantified by chemical diffusion modeling^13^,^14^,^15^,^16^. Such quantification however requires a comprehensive model of multicomponent melt diffusion and a knowledge of the advective history of the mingling magmatic system. The former is highly underconstrained by current models. The models that have been proposed to account for the multi-component nature of diffusion processes in igneous systems^16^,^17^,^18^ are typically based on the modeling of three or four components. Natural silicate melts contain far more relevant chemical species. The lack of a general model, accounting for the role of all chemical components and their interdependence in dynamic systems, significantly hinders our understanding of magmatic systems. The latter is an even greater problem as the advective history of mingling magmas is a chaotic process which cannot be unravelled by attempting to numerically “undo” the process of magmatic flow. The new methods presented here, based on the concept of the time decay of the variance of chemical composition during mingling, frees us from both of the above constraints in estimating the timescale of the mingling process.

## Time-series magma mixing experiments

**Figure 1:**
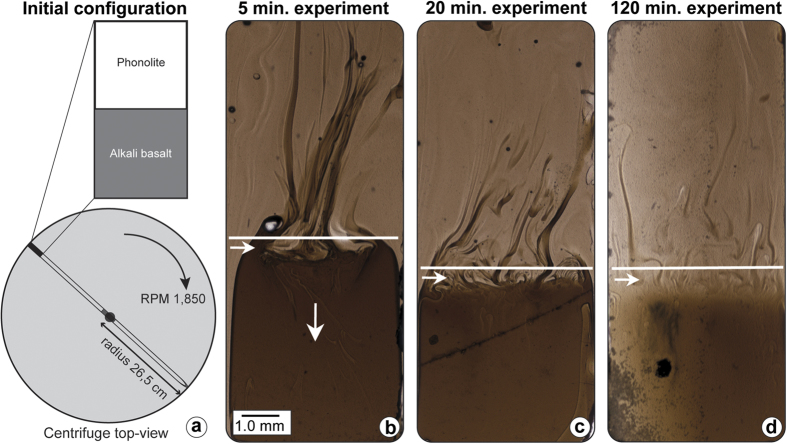
(**a**) Sketch of the centrifuge used to trigger the injection of the basaltic melt in the phonolite at 1,200 °C. The location and geometry of the experimental sample before the starting of the experiments are also shown. (**b**–**d**) Resultant glasses from magma mixing experiments at different mixing time *t* = 5 min (**b**), *t* = 20 min (**c**) and *t* = 120 min (**d**) The vertical white arrow in (**b**) indicates the direction of motion of the basaltic melt during injection. White lines correspond to the transects along which geochemical analyses have been performed and are located at approximately the division line of the two melts at *t* = 0. The horizontal white arrows in (**b**–**d**) indicate the direction in which the analyses were performed.

Here we perform a novel assessment of element mobility and chemical homogenization in silicate melt systems via the concept of Concentration Variance Decay (CVD). This new analysis is applied here to time-series experiments performed on mixing volcanic melts using a high-temperature (1,200 °C) centrifuge furnace (Fig. 1).

The initial compositions of end-members were an alkali basalt and a phonolite from the Campi Flegrei volcanic system (Italy)^19^. The centrifugal force exerted by rotation has accelerated the injection of the basalt into the phonolite (see Methods section). During injection tendrils of basalt are entrained into the phonolite triggering a “fountain-like” process of mingling. This configuration of the experimental sample and the resulting mixing dynamics are intended to simulate the triggering of magma mixing in nature via injection of mafic magmas into felsic magma chambers^20^,^21^. The time evolution of the mixing process results in the progressive mixing of the tendrils of basaltic melt with the phonolitic melt (Fig. 1).

**Figure 2:**
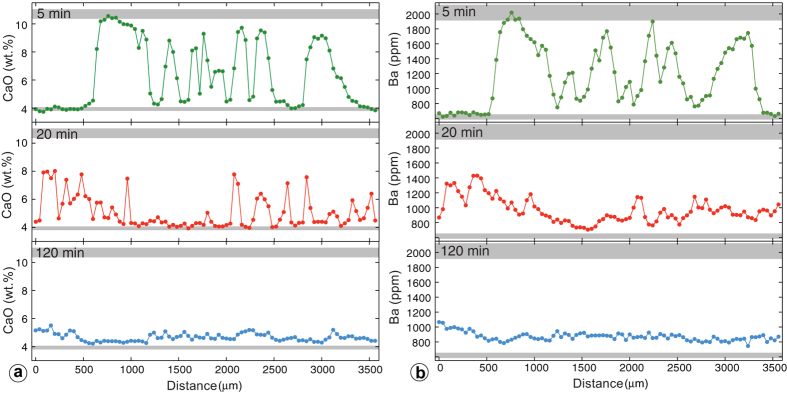
Representative compositional variation of CaO (**a**) and Ba (**b**) along the mixing interface (i.e. along the white horizontal lines in Fig. 1b-d) at different mixing times. Concentrations of initial basaltic and phonolitic melts are marked in grey areas including analytical uncertainties as standard deviations.

The mixing patterns observed may be described as filaments, swirls and bands ranging down to the micron length scale. These morphologies and length scales are topologically similar to those observed in natural rock samples where the mingling has been frozen-in^4^. The mixing process results in a transition from initially sharp chemical fluctuations oscillating between the two end-member compositions (Fig. 2) through progressive reduction of the amplitude and variability of these chemical fluctuations, to a final homogeneous hybrid composition (Fig. 2 and Supplementary Dataset).

**Figure 3:**
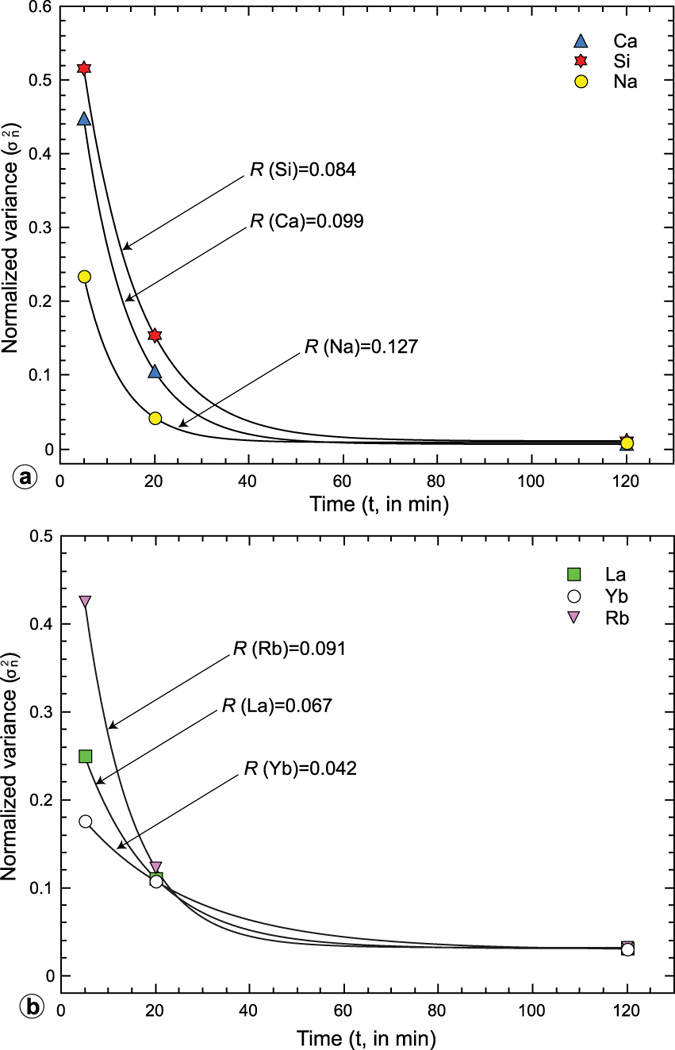
Concentration Variance Decay (CVD) for some representative major (SiO_2_, CaO, Na_2_O) oxides (**a**) and trace elements (Yb, La and Ba) (**b**) fitted using the equation reported in the text. The *R* value (i.e. the rate of CVD) is reported for each element.

We evaluated the degree of homogeneity of the mixture using the normalized concentration variance


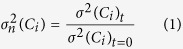


where 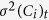 and 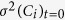 are the concentration variances of a given chemical element (*C*_*i*_) at time *t* (corresponding to the duration of the experiment) and time *t* = 0, respectively. Specifically, we calculated sample variance for the transects and population variance for the end-members; this procedure has been used consistently for both experimental and natural samples (see below). We observe that 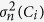 decreases rapidly, relaxing towards zero within 120 minutes (Fig. 3). The Concentration Variance Decay (CVD) for all chemical elements was fitted using the Matlab^®^ software by an exponential function of the form


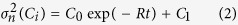


where *C*_0_, *R* and *C*_1_ are fitting parameters and *t* is the mixing time. The values of fitting parameters are reported in Table 1. The rate *R* at which concentration variance decays (CVD-*R*) is a metric quantifying element mobility during mixing (Fig. 3).

## Discussion

**Figure 4:**
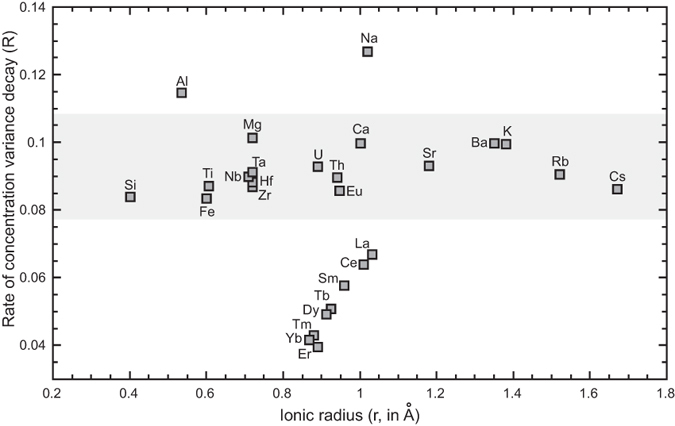
Estimated values of the rate of CVD (*R*) for all analyzed chemical elements plotted against their ionic radius. The range between 0.8 and 1.1, where *R* values for most elements are relatively similar, is highlighted in gray.

In Fig. 4 the estimated values of the rate of CVD (*R*) for all analyzed chemical elements are plotted against their ionic radii. Several observations can be made. Na shows the highest mobility, followed by Al. The other major elements (i.e. Mg, Ca, K, Si, Ti and Fe) show *R* values in the range 0.11–0.08. That same interval of *R* values is exhibited by several trace elements such as Nb, Ta, Zr, Hf, U, Th, Eu, Sr, Ba, Rb and Cs. Rare Earth Elements (REEs) display a distinct behavior: i) their *R* values are lower and ii) *R* decreases progressively with ionic radius. The exception to this trend is Eu whose *R* is comparable with the other elements (Fig. 4).

It is somewhat instructive to observe correlations between the results of the CVD-*R* and published tracer or self-diffusion data. Where data exist, the mobilities tracked in this study can be compared with coefficients of diffusivity in multicomponent systems. Note that as of yet virtually no systematic studies report the diffusion coefficients for the complete set of relevant major and trace elements in natural melts, and thus our comparison is, at best, a partial one. The major alkalis (i.e. Na and K) have much larger diffusion coefficients relative to the other major elements^13^,^14^. Our results corroborate this behavior for Na, but not for K. The melts analyzed in Baker^13^,^14^ have maximum K_2_O contents of ca. 4%. The content of K_2_O in the melts from Campi Flegrei used in our experiments is higher (up to ca. 8%). It has been shown^13^ that the diffusion coefficient of a chemical element has the tendency to decrease with the abundance of the same element in the melt. This may explain why K displays a lower mobility than Na in our melts. As for the other major elements, the limited variation in *R* reflects similar mobility (Fig. 4), in agreement with known diffusivities^13^,^14^.

Systematic studies of trace element diffusivities are few^22^,^23^,^24^. Available data indicate that Rb, Sr and Ba have the highest values of diffusivities; whereas diffusivity values for REEs are lower^22^,^23^,^24^, in agreement with our results (Fig. 4). However, for the case of element diffusivity in jadeitic melts, U and Th have been shown to have the lowest diffusivities^24^, contrary to what we observe. Nb, Ta, Zr, and Hf, are generally regarded as slowly diffusing species^22^,^24^. In apparent contrast our results demonstrate that their high *R* values are similar to those estimated for Th, Sr, or Rb. The systematic decrease of *R* values from light to heavy REEs is in general agreement with literature data^22^,^24^. As for Eu, it can be present in the divalent state in silicate melts, while the other REEs occur only in the trivalent state. This presumably yields a larger mobility for Eu^22^,^24^.

The above analysis illustrates that, whereas some elements exhibit similarities between *R* and the diffusion coefficient, others do not. This fact highlights that diffusion coefficients estimated for individual melts can in general not be robustly applied to the study of the space and time complexity generated by mixing processes. In contrast, the use of CVD-*R* values incorporates, in a single variable, the sum of a range of processes that affect element mobility, including: 1) partitioning of chemical elements into structurally different melts^25^, 2) the dependence of diffusivities on multicomponent composition^16^, 3) the influence of advection on apparent diffusive fluxes^26^ and 4) the potential development of “uphill” diffusion patterns^15^. Thus the variable *R* provides a first order cumulative assessment of element mobility. It does not of course identify the dominant mechanisms driving elemental diffusion. The understanding of the interplay of all the variables influencing element mobility will likely require combined thermodynamic- and fluid dynamic-based numerical simulations in future.

### Estimate of mixing-to-eruption time

We now apply the CVD-*R* method to the Campi Flegrei system in order to estimate the timescales of magma mixing immediately preceding eruptions (i.e. the mixing-to-eruption time interval). Note that the rationale we employ here (using experimental data to make inferences about natural processes) is based on the consideration that the space and time evolution of magma mixing processes is governed by the development of chaotic dynamics. This assertion is well-substantiated in previous studies employing natural samples^4^,^27^, numerical simulations^28^,^29^ and experiments^7^,^30^. The stretching and folding dynamics of fluid elements, responsible for the development of chaotic dynamics, are also responsible for the production of scale-invariant fractal structures. This results in the generation of self-similar compositional domains propagating in the magmatic mass over several orders of magnitude^28^,^31^. It has, for example, been demonstrated that compositional heterogeneity generated by magma mixing propagates from the meter to the micron length-scale^4^. It follows that magmatic systems can be analyzed at any length scale and they will provide, statistically, the same type of information. Importantly, this justifies the use of smaller experimental samples in the effort to explain natural processes on a much wider range of scales.

**Figure 5:**
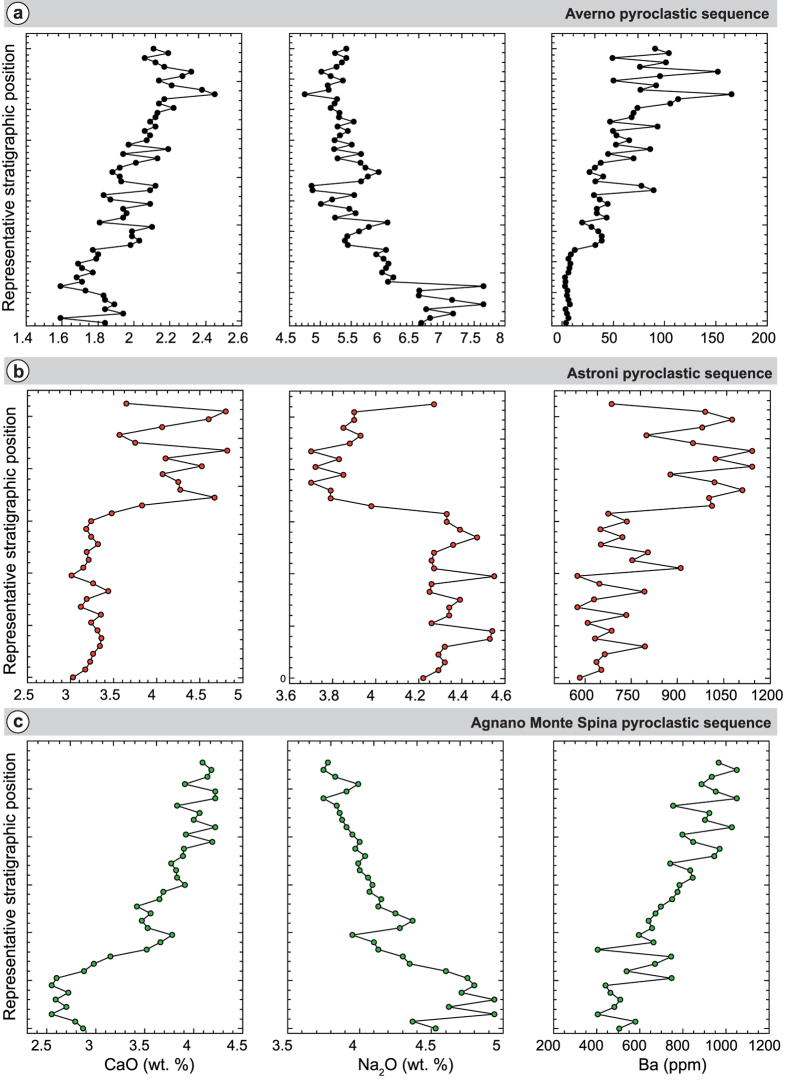
Variation of some representative major and trace elements across the pyroclastic sequences of Averno (**a**), Astroni (**b**), and Agnano Monte Spina (**c**) used to estimate the mixing-to-eruption time with the CVD method. Height of the sequence is ca. 20 m., 16 m. and 15 m. for Averno, Astroni, and Agnano Monte Spina, respectively.

The concentration variance [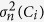] was calculated (Table 1) for each analyzed chemical element in three pyroclastic sequences which correspond to three explosive eruptions (Averno, Astroni and Agnano-Monte Spina) of the Campi Flegrei volcanic system (Italy)^19^ where magma mixing is extraordinarily well-documented^32^. The three sequences are geochemically zoned (Fig. 5) indicating the occurrence of compositional gradients in these volcanic systems and highlighting the contemporaneous presence of melts with different compositions (with more mafic magmas underlying more evolved magmas) (see Supplementary Dataset). Small-scale compositional fluctuations (of the order of a few tens of centimeters) along the pyroclastic sequences also provide evidence for the action of mixing dynamics acting in the plumbing systems before the onset of eruptions. The plots display the current position of samples in the stratigraphic sequences (so that they need to be reversed to visualize the chemical zoning in the original magma reservoirs). To estimate the mixing time we first calculated the variance 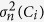 values for each chemical element measured on natural samples (see Methods section; Table 1). Next, using the exponential empirical relationships derived from experiments for each element, the mixing time was estimated (Table 1). Results indicate that mixing lasted for 18 ± 5 (s.d.), 13 ± 4 (s.d.) and 15 ± 4 (s.d.) minutes for Averno, Astroni and Agnano-Monte Spina events, respectively. These estimates are statistically robust as they are based on a large number of chemical elements converging to short timescales. We nevertheless further tested the robustness of our statistical analysis by randomly removing from the pyroclastic sequences a progressively increasing number of samples. Results indicate that timescales can be estimated with an uncertainty comparable to the uncertainty resulting from averaging the time estimates for all chemical elements (i.e. of the order of  ± 4–5 min s.d.). This holds for removing up to 75% of the data from the three stratigraphic sections. If a larger percentage of samples is removed, time estimates start to fluctuate as the number of samples become subcritical with respect to representativeness. This implies that, for example, in the case of the Averno pyroclastic sequence, collecting about 15 samples in the stratigraphic section is enough to obtain robust mixing-to-eruption timescales.

Previously^33^ the Averno and Astroni events were suggested to have mixing-to-eruption times on the order of days. These times are now shifted to tens of minutes on the basis of our experimental calibration. We believe that these new timescales are more realistic. In particular, previous mixing experiments were performed by placing a phonolite on the top of a trachyte and stirring using a spindle vertically positioned into the crucible^33^. Given the gravitationally stable configuration (denser trachyte at the bottom and lighter phonolite at the top) there was little chance for mixing. In particular, mixing occurred essentially at the interface between the two melts by diffusion. This was reflected in longer timescales. The new experiments presented here were performed by injecting the basalt into the phonolite. This is considered as the main mechanism for the replenishment and mixing in natural magma chambers^20^,^21^. This process generated a large amount of contact interfaces between the two melts, increasing chemical exchanges, as observed in several natural samples showing magma mixing patterns^4^,^28^,^34^. In addition, these two compositions have been envisaged as the most likely end-members participating to magma mixing processes in the Campi Flegrei volcanic area^35^. Therefore, we are prone to believe that, given the more realistic conditions of the new experiments, these timescales are more robust than previous one.

Timescales obtained in this work indicate that very little time elapsed from the moment mixing started to eruption. These results are in agreement with recent numerical simulations of magma mixing^36^ that highlight mixing timescales of a few hours to attain complete hybridization of magmas for the Campi Flegrei magmatic systems. Our results constrain mixing durations before eruption to tens of minutes. Establishing whether the mixing process caused the eruptions or vice versa is not easy (for example the ascent of magmas towards the Earth’s surface may have provoked mixing of stratified plumbing systems). In both cases, however, estimating the mixing-to-eruption duration is highly valuable as it provides unprecedented information about the timing elapsing between the beginning of instability in the plumbing system and the eruption. Estimates of the location of the shallow magma reservoirs that were involved in the recent eruptive activity of the Campi Flegrei volcanic systems studied here indicate depths around four to five kilometers^37^,^38^. This implies average ascent velocities of the magma of the order of 5–8 meters per second and point to very rapid magma ascent through the sub-volcanic systems. These ascent velocities are of the same order of magnitude as those recently estimated, for example, for calc-alkaline rhyolitic magmas (ca. 1 m/s)^39^. The slightly higher ascent velocity values derived here can possibly be explained by the fact that K-alkaline magmas from Campi Flegrei have lower viscosities compared to calc-alkaline rhyolitic magmas. The higher alkali contents lead to a strongly reduced viscosity of Campi Flegrei magmas favoring higher ascent velocities. These ascent velocities are also very close to the fragmentation front velocities achievable due to decompression fragmentation, a necessary condition for sustained eruptive flow^40^. In addition, and perhaps most importantly, these magma bodies experienced mixing processes: the introduction of hot and low viscosity mafic melts into higher viscosity melts may have promoted fluidization of the magmatic mass^12^ and heating due to the retrograde solubility of H_2_O^41^,^42^, further accelerating their migration towards the surface.

These results have implications for civil protection planning of future volcanic crisis because these high velocities of ascending magmas may imply little warning in volcanic crises.

It is important to note that the method we propose to estimate the mixing-to-eruption timescale has been tested using end-members from the Campi Flegrei and applied to the same volcanic system. If the end-member compositions are changed, the rheological properties and the relative mobility of the elements can change. Substantially different end-member couples require new experiments to derive empirical relationships to be used to infer the mixing-to-eruption timescales for other magmatic/volcanic systems.

We anticipate our findings to be a starting point towards a unifying model explaining chemical exchanges in magmatic systems and supplying information on the use of chemical element mobility as geochronometers for volcanic eruptions. This may provide unparalleled clues for building an inventory of past and recent volcanic eruption timescales and could be decisive for hazard assessment in active volcanic areas.

## Methods

Natural end-members used in the experiments come from Agnano Monte-Spina B1-B2 phonolitic tuff and the glass matrix of the alkali-basalt from Minopoli (Campi Flegrei volcanic area, Italy)^43^. These compositions are considered as suitable end-members for magma mixing processes in the study area^35^. The values of measured viscosity and calculated density of the basaltic and the phonolitic melt at 1200 °C are ca. 200 and 20000 Pas, and 2.82 and 2.50 g/cm^3^, respectively. Starting materials for the experiments have been prepared by melting these natural products. End-members were completely homogenized at 1,400 °C in a NaberthermTM furnace for 24 hours. Successively, the homogeneity and the absence of crystals in the samples were tested. Glass cylinders with 0.5 cm height and 0.5 cm diameter were drilled, polished on both sides, and mounted, one on top of the other, inside a Pt-capsule (diameter, d = 0.5 cm; length, l = 1.0 cm). The Pt-capsule was sealed on the top and the bottom leaving a small (ca. 0.5 mm) aperture to allow for eventual gas release. The relative mass fractions of the phonolitic and basaltic melts for the experiments were 50%–50%.

The centrifuge used to perform the experiments is a Cryofuge 8500i (Heraeus InstrumentsTM) modified to host a high-temperature furnace (up to 1,200 °C). Three Pt–PtRh10 (type S- Kanthal™ wire) thermocouples were used to monitor the temperature at the top, bottom and the middle of the Pt-capsule containing the samples. A slip-ring assemblage provided the electric power supply and thermocouple reading from the furnace. Sliding electrical contacts (copper rings and brass-carbon brushes from Kontakt-Kohlen Kever, Koln) of the slip-ring assemblage consist of a rotating central section (rings) and a stationary outer section (brushes). More details about the machine are reported in Dorfman *et al.*^44^. Before running the experiment the slip-ring assemblage was mounted on the shaft of the centrifuge. This machine was used in the past for different purposes (e.g. measurements of viscosity using the falling-sphere method, experiments of unmixing using basaltic magma) and detailed technical information are reported in Dorfman *et al.*^44^ and Veksler *et al.*^45^.

The Pt-capsules containing the end-members were then introduced into the outer container of the centrifuge. The same conditions were used for all experiments. A rotation speed (*n*) of 1850 rpm was used to perform experiments at the temperature of 1,200 °C. We performed three experiments at different times: 5, 20 and 120 minutes.

Experiments were performed at Reynolds number (Re) of the order of 10^−10^. Experiments, therefore, were performed under laminar fluid dynamics conditions as these conditions are likely to prevail in magmatic systems^46^,^47^. Re was calculated as reported in Yuan and Zheng^48^.


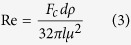


where *F*_*c*_ = *ρVω*2*R*_*c*_ is the centrifugal force, *ρ* is density, *V* is the volume of the capsule, *ω* is the angular velocity of the centrifuge, *R*_*c*_ is the distance between the experimental capsule and the center of rotation of the centrifuge (i.e. 26.5 cm), *l* and *d* are the length and the diameter of the capsule, and *μ* is viscosity. Average values of density (*ρ*) and viscosity (*μ*) were calculated considering the initial proportions of end-members (i.e. 50%–50%).

At the end of experiments the samples were quenched in air by turning off the centrifuge rotation and the heating. During quenching, the temperature of the sample dropped from 1,200 to 1,100 °C in 10 s, from 1,100 to 1,000 °C in 15 s; further cooling to 800 °C required approximately 1.5–2 minutes. The experimental samples were then cut in the middle part and prepared for optical inspections, electron microprobe and ICP-MS-Laser Ablation analyses.

Major and trace elements were measured on an average of 100 data points along each electron microprobe at the Department of Earth and Environmental Sciences, LMU-Munich. Analytical conditions were: 15 kV acceleration voltage and 20 nA beam current. In order to avoid alkali loss during analyses a defocused 10-μm beam was used. Synthetic wollastonite (Ca, Si), periclase (Mg), corundum (Al), natural orthoclase (K), and albite (Na) were used as standards. The PAP procedure^49^ was used to perform the matrix correction. For all analyzed elements standard deviations were lower than 2.5%. Trace elements determinations were performed using the Laser Ablation ICP-MS microanalysis facility installed at the Department of Physics and Geology of the University of Perugia (Italy)^50^. Analyses were performed using a spot size of 40 μm; consecutive data points were analyzed with a spacing of 40 μm. This allowed us to appreciate in details the compositional variability in the studied experiments. Analytical precision was better than 10% (s.d.) for all analyzed elements^51^.

The total number of analyzed natural samples for Astroni (Astroni 6 eruption)^33^, Averno (Averno 2 eruption)^33^, and Agnano-Monte Spina^37^ pyroclastic sequences was 36, 62 and 38, respectively. Samples are mostly glassy with a few crystals of ca. 6%, 3%, and 4% for Astroni, Averno, and Agnano-Monte Spina, respectively. We consider this crystal contents having a negligible effect on the chemical variability of natural rocks compared to the variability triggered by the magma mixing process. Each sample consists of several (10-15) pumice fragments collected in the same stratigraphic level^33^. Details on sample preparation for geochemical analysis are given in Perugini *et al.*^33^. Major elements were analyzed by combined X-ray fluorescence and wet chemical techniques. Trace elements were analyzed by LA-ICP-MS graphite electrode tetraborate fusion by using the method described in Petrelli *et al.*^50^,^51^ at the Department of Physics and Geology, University of Perugia (Italy). Normalized concentration variance for each element used in the estimate of mixing-to-eruption timescale was calculated as the ratio between the concentration variance among all samples and the variance between the two most extreme compositions found in the stratigraphic sequence.

## Additional Information

**How to cite this article**: Perugini, D. *et al.* Concentration variance decay during magma mixing: a volcanic chronometer. *Sci. Rep.* **5**, 14225; doi: 10.1038/srep14225 (2015).

## Supplementary Material

Supplementary Dataset 1

Supplementary Dataset 2

## Figures and Tables

**Table 1:** Fitting coefficients (*C*_*0*_, *R*, *C*_*1*_) used to fit the decay of concentration variance for the chemical elements measured on experimental samples and used to estimate the mixing-to-eruption time for the three pyroclastic sequences of Averno, Astroni e Agnano Monte Spina (AMS).

	***Fitting parameters***			***Variance***			***Time (min)***		
	***C*_*0*_**	***R***	***C*_*1*_**	***Averno***	***Astroni***	***AMS***	***Averno***	***Astroni***	***AMS***
SiO_2_	0.769	0.084	0.011	0.122	0.187	0.139	23	18	21
TiO_2_	0.529	0.087	0.014	0.115	0.122	0.146	19	18	16
Al_2_O_3_	0.684	0.115	0.017	0.096	0.181	0.103	19	12	18
FeO	0.615	0.083	0.016	0.071	0.206	0.139	29	14	19
MgO	0.858	0.101	0.007	0.129	0.180	0.155	19	16	17
CaO	0.728	0.100	0.007	0.103	0.186	0.223	20	14	12
Na_2_O	0.425	0.127	0.009	0.102	0.194	0.161	12	7	8
K_2_O	0.793	0.100	0.010	0.145	0.193	0.111	18	15	21
Rb	0.640	0.091	0.018	0.102	0.205	0.138	23	14	18
Sr	0.494	0.093	0.018	0.087	0.201	0.164	21	11	13
Zr	0.476	0.087	0.009	0.160	0.162	0.164	13	13	13
Nb	0.475	0.088	0.015	0.155	0.227	0.150	14	9	14
Ba	0.385	0.100	0.008	0.070	0.202	0.170	18	7	9
La	0.306	0.067	0.031	0.102	0.145	0.155	22	15	13
Ce	0.229	0.064	0.041	0.122	0.179	0.131	16	8	15
Sm	0.209	0.058	0.063	0.099	0.133	0.137	31	19	18
Eu	0.212	0.086	0.031	0.156	0.135	0.137	6	8	8
Tb	0.141	0.051	0.044	0.103	0.141	0.133	17	7	9
Yb	0.181	0.042	0.029	0.117	0.148	0.142	17	10	11
Hf	0.478	0.090	0.030	0.165	0.131	0.156	14	17	15
Ta	0.636	0.091	0.021	0.170	0.147	0.172	16	18	16
Th	0.582	0.090	0.011	0.135	0.153	0.172	17	16	14
U	0.526	0.093	0.013	0.150	0.156	0.152	14	14	14

The variance calculated for the same elements on the three pyroclastic sequences and the corresponding timescale (in minutes) are also reported. The timescale of each eruption is the average values of the timescales measured for each element (i.e. 18 ± 5 (s.d.), 13 ± 4 (s.d.) and 15 ± 4 (s.d.) minutes for Averno, Astroni and Agnano-Monte Spina, respectively).
